# Serine Metabolic Reprogramming in Tumorigenesis, Tumor Immunity, and Clinical Treatment

**DOI:** 10.1016/j.advnut.2023.05.007

**Published:** 2023-05-13

**Authors:** Wang Shunxi, Yuan Xiaoxue, Song Guanbin, Yang Li, Jin Junyu, Liu Wanqian

**Affiliations:** 1 Key Laboratory of Biorheological Science and Technology, Ministry of Education, Bioengineering College, Chongqing University, Chongqing, China; 2 Department of Oncology, Chenjiaqiao Hospital, Shapingba, Chongqing, China

**Keywords:** serine metabolism, tumorigenesis, tumor immunity, tumor stem cell, therapeutic targets

## Abstract

Serine has been recently identified as an essential metabolite for oncogenesis, progression, and adaptive immunity. Influenced by many physiologic or tumor environmental factors, the metabolic pathways of serine synthesis, uptake, and usage are heterogeneously reprogrammed and frequently amplified in tumor or tumor-associated cells. The hyperactivation of serine metabolism promotes abnormal cellular nucleotide/protein/lipid synthesis, mitochondrial function, and epigenetic modifications, which drive malignant transformation, unlimited proliferation, metastasis, immunosuppression, and drug resistance of tumor cells. Dietary restriction of serine or phosphoglycerate dehydrogenase depletion mitigates tumor growth and extends the survival of tumor patients. Correspondingly, these findings triggered a boom in the development of novel therapeutic agents targeting serine metabolism. In this study, recent discoveries in the underlying mechanism and cellular function of serine metabolic reprogramming are summarized. The vital role of serine metabolism in oncogenesis, tumor stemness, tumor immunity, and therapeutic resistance is outlined. Finally, some potential tumor therapeutic concepts, strategies, and limitations of targeting the serine metabolic pathway are described in detail. Taken together, this review underscores the importance of serine metabolic reprogramming in tumorigenesis and progression and highlights new opportunities for dietary restriction or selective pharmacologic intervention.

**Statement of Significance**

This article focuses on an important gap of serine metabolism research—the interactions of serine metabolic reprogramming in tumor and potential associated therapeutic benefits. Mechanisms such as oncogenesis, progression, and therapeutic resistance and immune relevance by serine are particularly highlighted.

## Introduction

Metabolic reprogramming is an important hallmark of malignant tumor progression [[1], [2], [3]]. Cancer cells maintain their survival and rapid proliferation through metabolic reprogramming, which can provide a large amount of energy and macromolecular substances required for metabolic conversion [4]. Compared with normal cells, most cancer cells provide energy for themselves through glycolysis [5]. Owing to its low productivity, many cancers increase the utilization of glucose and the absorption of amino acids, such as glutamine [6]. However, in rapidly proliferating cancer cells, high consumption of glucose and glutamine is insufficient to support the accumulation of biomass. Instead, nonglutamine amino acids provide most of the carbon and nitrogen units, such as serine, which is essential for cancer cell survival [7]. Serine derived from cellular glycolysis and exogenous uptake can be converted to glycine and provide 1-carbon units for 1-carbon metabolism [8]. Its metabolites can be used for various other biosynthetic purposes, such as energy production, the synthesis of nucleic acids and lipids, amino acid homeostasis, epigenetic regulation, and maintenance of cellular redox state homeostasis in cancer cells [9,10]. In addition, elevated deoxysphingolipids by dietary serine restriction or inhibition of endogenous synthesis, relying on alanine as a substrate for the promiscuous serine palmitoyltransferase reaction, can promote cytotoxicity in neurons, mitigate cancer growth, and extend the survival of cancer patients [[11], [12], [13], [14], [15]]. Thus, understanding the myriad metabolic fates of serine in cancer is of key interest in the cancer biology field and target therapy.

The connection between serine metabolism and cancer was first suggested when it was observed that the activity of phosphoglycerate dehydrogenase (PHGDH) in passaged rat hepatoma cell lines was higher than that in normal rat liver cells [16]. Later, Snell et al. [17] found that PHGDH has relatively high activity in rat liver cancer tissues with a strong cell renewal ability and increased activity in neonatal and regenerating livers [18]. The activation of the PHGDH–phosphoserine aminotransferase 1 (PSAT1)–phosphoserine phosphatase (PSPH) pathway by several factors [eg, solute carrier family 2 member 5 (GLUT5), pyruvate kinase M2 (PKM2), activating transcription factor (ATF) 3/4, P53, and liver kinase B1 (LKB1)] promotes the synthesis of serine, which can direct the downstream serine glycine 1-carbon pathway (SGOCP), tricarboxylic acid cycle (TCA) pathway, and lactic acid and lipid synthetic pathways [8,9,12,[18], [19], [20], [21], [22]]. Moreover, various substances produced by serine catabolism not only can be raw materials for the synthesis of intracellular biomacromolecules but also can maintain the redox state of cells by producing surplus nicotinamide adenine dinucleotide (NADH) and support epigenetic changes [8,10,23]. It is beneficial to the development of multiple cancers, such as myeloma, squamous cell cancer, melanoma, and liver cancer [[24], [25], [26], [27], [28]]. Furthermore, increased serine and downstream 1-carbon pathway metabolism have also been implicated in the drug resistance and immune response of myriad tumor cells [11,[29], [30], [31], [32]]. Some novel therapeutic agents targeting serine metabolic pathways (e.g., PHGDH, PSAT1, and PSPH) in cancer seem to have great potential [33,34]. Although there is extensive documentation for serine metabolism in cancers and immune diseases, its significance in tumor progression, immunotherapy and its-related regulatory mechanisms, and the contribution of serine metabolism to cancer metabolic reprogramming pathways have not been fully appreciated.

**FIGURE 1:**
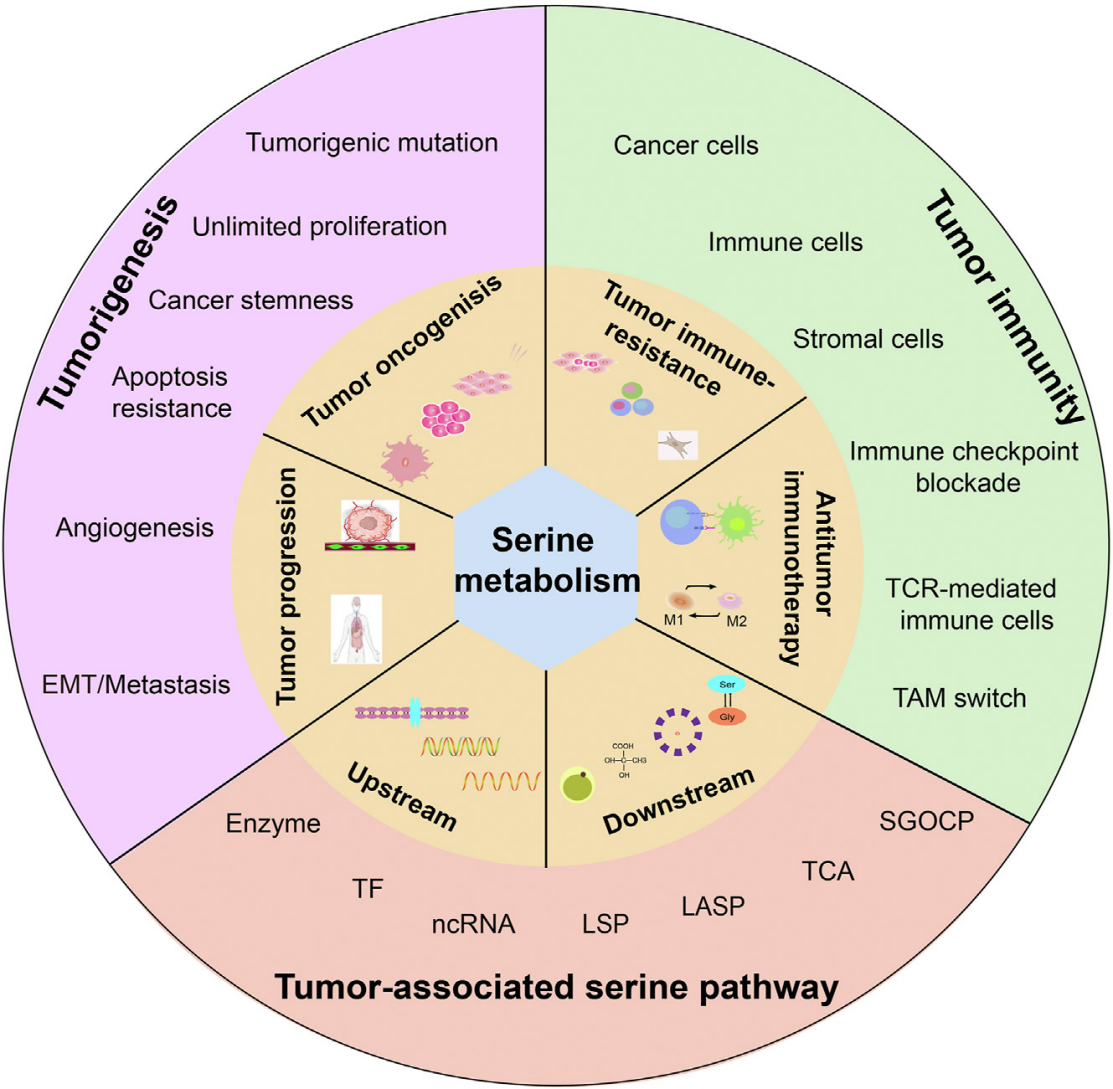
Serine is a key metabolite that connects tumorigenesis, tumor immunity, and target intervention. The activation of serine metabolism after underlying tumor microenvironment (TME) conditions or because of hyperactive sugar consumption promotes tumorigenesis and tumor immunity.

In this study, we have attempted to dissect the molecular mechanisms that target serine metabolic reprogramming in cancers. We also underscored the vital role of serine metabolism that connects tumorigenesis, progression, tumor immunity, and potential targeting by focusing on recent insight into serine function (Figure 1). Finally, we highlighted recent achievements and discussed the prospects of serine metabolism signaling as a therapeutic and complementary target for cancer treatment.

## Regulation Pathways of Serine Metabolism

### Serine anabolism

Increasing evidence shows that intracellular serine can be synthesized de novo by tumor cells or absorbed by serine transporters [35,36]. De novo synthesis of endogenous serine is mediated by PHGDH, PSAT1, and PSPH, using the glycolytic or gluconeogenic intermediate 3-phosphoglycerate (3-PG) as the substrate [8,37]. However, there are 2 views on how serine is synthesized. One group championed a nonphosphorylated pathway based on the observation that [^14^C] glyceric acid administered to rats gave rise to radioactive serine in tissues. Another group proposed that serine is derived from the glycolytic intermediate 3-PG thorugh a phosphorylated pathway. The phosphorylated pathway is now known as the physiologic route of serine synthesis, whereas the nonphosphorylated pathway represents a means of serine catabolism [38]. However, increased serine biosynthesis from glucose in tumor cells and tumor-associated cells is important for supporting the growth and therapy resistance of many cancers [8,9]. In addition, exogenous serine from the tumor environment is mainly transported by the extracellular transporters alanine–serine–cysteine transporters 1 and 2, which are elevated in a variety of tumors and are valuable prognostic markers for patients, such as lung cancer, ovarian cancer, breast cancer, hepatocellular carcinoma, and colorectal adenocarcinoma [[38], [39], [40]]. Thus, understanding the origin and synthesis mechanism of serine in cancer cells is important for therapeutic intervention in cancers and remains to be clarified.

#### Serine biosynthesis in cancer cells and cancer-associated cells

Emerging evidence shows that serine biosynthesis is mainly activated by transcription factors or other mediators in rapid-growth cancers [8,[9], [18]]. Previously, ATF3 and ATF4 were identified as robust master transcription factors that directly bind to the promoter region of serine biosynthesis pathway (SSP) genes to promote serine biosynthesis and multiple tumor cell growth (e.g., sarcoma cells, prostate cancer cells, and HCT116 colon cancer cells) [13,41]. It is also well documented that mediators, such as nuclear factor erythroid-2 related factor 2, sirtuin 2 (SIRT2), protein kinase C (PKC) λ/ι, and Tat-interacting protein 60, mediate serine synthesis by upregulating or downregulating the expression of PHGDH or other metabolic genes in various cancer cells [8,[42], [43], [44], [45]]. These results indicate that serine anabolism in cancer cells is orchestrated by multiple signaling factors or mediators, and the underlying mechanism remains obscure.

In addition to cancer cells, tumor-associated cells, such as M2-like tumor-associated macrophages (TAMs), bear the highest individual capacity to take up intertumoral glucose, indicating that they are a major contributor to tumor glycolysis-derived serine production [46]. On immune-activated CD8^+^ CTLs (cytotoxic T-lymphocytes), the activation of c-myc and hexokinase 2 (rate-limiting enzyme for glycolysis) induced by mammalian target of rapamycin (mTORC) 1 increases glucose uptake and glycolytic activity, resulting in an increased serine synthesis [47]. However, knockout of coactivator-associated arginine methyltransferase 1 significantly decreases serine synthesis by reducing pyruvate kinase (PK) activity in fibroblasts [48]. Previous studies have shown that altering tumor environment factors (e.g., matrix stiffness) directs TAMs and neutrophil polarization, T-cell differentiation, and cell glycolysis [[49], [50], [51]]. Thus, it is reasonable to speculate that increasing the extracellular matrix stiffness of the tumor environment during solid tumor progression may enhance glycolysis-derived serine biosynthesis. These results suggest that glycolysis-derived serine biosynthesis from tumor-associated cells plays a vital role in tumorigenesis and progression and is worth further elucidation.

#### Regulation of serine synthesis

*Glycolytic pathway.* As a side branch of glucose metabolism, serine synthesis has extensive interaction with the glycolytic pathway. Le Douce et al. [52] proposed that astrocytes have impaired glycolysis flux and produce less glucose-derived serine. GLUT5, a glucose and fructose transporter, has been reported to improve glycolysis flux by increasing the absorption of fructose to expedite the synthesis of serine in the cytoplasm [20]. However, it is not clear whether it regulates serine synthesis as a glucose transporter or a metabolic mediator. Moreover, the key enzymes in the glycolytic pathway, glyceraldehyde-3-phosphate dehydrogenase (GAPDH) and PKM2, can regulate the synthesis of serine in different ways [48,53,54]. GAPDH has been reported to promote the diversion from glycolysis to serine biosynthesis by increasing histone methylation concentrations [53]. As a natural ligand, serine can affect the activity of PKM2. Low serine concentrations in tumor cells inhibit PKM2 activity, resulting in the accumulation of 3-PG production to promote serine synthesis [48,54].

Previous studies reported that glycolysis is one of the major pathways of NADH production to maintain the redox state in tumor cells. Part of the NADH produced by GAPDH in glycolysis needs to be converted to NAD^+^ by lactate dehydrogenase in the cytosol to maintain the activity of GAPDH and promote the glycolytic pathway [55,56]. A recent study showed that in proliferating tumor cells, NADH produced by the glycolytic pathway is first shuttled into mitochondria. When the shuttle is oversaturated, NADH is used by lactate dehydrogenase to convert pyruvate into lactate. Conversely, the catabolic pathway of glucose-derived serine in mitochondria also produces NADH. Serine-derived NADH is the main source of intracellular surplus NADH production in the presence of impaired respiration [57]. Therefore, it is reasonable to speculate that the increase in glucose-derived serine may be an attempt to produce more mitochondrial NADH in the presence of shuttle limitations to reduce carbon waste through fermentation.

##### Serine synthetic pathway

The glycolytic intermediate 3-PG is the major entry substrate of the serine synthetic pathway (SSP) and is metabolized through a series of biochemical reactions by 4 cytoplasmic enzymes, PHGDH, PSAT1, PSPH, and serine hydroxymethyltransferase (SHMT) 1, and 1 mitochondrial enzyme, SHMT2, into serine. Some transcription factors and mediators have been reported to mediate the synthesis of serine by activating synthetic enzymes.

ATF4, a key transcription factor for adaptation to cellular stress, upregulates the gene expression of all 3 enzymes in serine biosynthesis: PHGDH, PSAT1, and PSPH. It can also activate the expression of downstream SGOC metabolism genes such as SHMT2 and methylenetetrahydrofolate dehydrogenase (MTHFD) 2 [8]. ATF4 is also required for H3K9 methyltransferase euchromatic histone lysine methyltransferase 2 (G9A) and H3K9 demethylase lysine demethylase 4C to transcriptionally activate serine biosynthesis [58,59]. Moreover, ATF4 itself is the target gene of other transcription factors, such as ATF3, nuclear factor erythroid-2–related factor 2, myelocytomatosis oncogene (MYC), and tumor protein p53 (P53). Moreover, tumor-related protein p73 (TRP73), hypoxia-inducible factor (HIF)-1, specificity protein 1, and nuclear transcription factor Y have been reported to induce the expression of PHGDH, PSAT1, and PSPH, which contribute to regulating serine biosynthesis and metabolism in cancer cells [[18], [60],^61^]. In addition, linc01564, as a long noncoding RNA in response to glucose deprivation by ATF4, induces the expression of PHGDH and promotes serine biosynthesis [28]. Further studies are necessary to characterize the effect of tumor environmental factors on ATF4-dependent and ATF4-independent serine biosynthesis [62].

Aside from transcription factors, adenomatous polyposis coli (APC), cullin 4A, alcohol dehydrogenase 1C, protein kinase Cλ/ι, parkin, and RING-finger protein 5 have also been reported to influence serine synthesis. For example, mutation of the adenomatous polyposis coli gene activates Wnt/β-catenin signaling, leading to enhanced expression of genes related to serine synthesis [30,63]. CUL4A promotes the activity of PHGDH through monoubiquitination of PHGDH, resulting in an increase in serine levels [64]. Elevated alcohol dehydrogenase 1C reduces the expression of PHGDH/PSAT1 and the serine concentration [65]. By contrast, downregulation of protein kinase Cλ/ι in de novo and therapy-induced neuroendocrine prostate cancer results in the upregulation of serine biosynthesis through an mTORC1/ATF4–driven pathway [44]. In addition, PHGDH ubiquitination by parkin or RING-finger protein 5 inhibits serine synthesis [45,66].

Recent studies have reported that Kirsten rat sarcoma viral oncogene homolog (Kras) mutation promotes *SSP* gene expression and resistance to serine starvation in pancreatic cancer [62]. The mutation of Kras is usually accompanied by loss of LKB1 in tumor cells [[67], [68], [69]]. Loss of LKB1 in mutant KRAS^G12D^ tumor cells modulates the expression of PSAT1 and PSPH in an mTOR-dependent manner [70,71]. Furthermore, in tuberous sclerosis complex–deficient cells, the increase in the oncogene interleukin (IL)-6 induces PSAT1 expression and serine anabolism [72].

### Regulation of serine catabolism

Serine affects downstream metabolic pathways in different ways, such as the SGOCP, TCA cycle, lactic acid, and lipid synthetic pathways [8,9,12,21,22]. The changes in these metabolic pathways are closely related to energy metabolism, macromolecular biosynthesis, redox state balance, epigenetic regulation, and other processes in tumor cells and tumor-associated cells.

#### Serine glycine 1-carbon pathway

Serine catabolism is initiated by SHMT1/2 (1 and 2 represent the site of the reaction: the cytoplasm or mitochondria, respectively), involving glycine and 1-carbon units, which is called the SGOCP [10,23]. One-carbon metabolism includes the bicyclic pathway formed by the coupling of folate and methionine cycles and the transsulfuration pathway [10,23,73].

In the folate cycle, folate is reduced by dihydrofolate reductase and finally converted to tetrahydrofolate (THF). THF accepts a 1-carbon unit, which is formed in the process of transforming serine into glycine catalyzed by SHMT1/2 and is converted into 5,10-methylene-THF. Then, methylene-THF is converted to 10-formyl-THF by MTHFD 1/2/L or 5-methyltetrahydrofolate (mTHF) by methylenetetrahydrofolate reductase (MTHFR). This process is accompanied by the production of nicotinamide adenine dinucleotide phosphate oxidase (NADPH) in the cytoplasm and NAD(P)H in mitochondria. Then, mTHF can be demethylated again and converted back to THF by methionine synthase.

Demethylation of mTHF completes the folate cycle and begins the methionine cycle. mTHF transfers carbon units to homocysteine, which in turn is converted to methionine-by-methionine adenylyl transferase. Next, methionine is used to generate S-adenosylmethionine (SAM), a substrate for methylation reactions, which forms S-adenosylhomocysteine when demethylated. The latter is catalyzed by S-adenosylhomocysteine hydrolase to homocysteine, thus completing the entire methionine cycle. The methionine cycle not only is necessary for protein synthesis but also provides a methyl donor by SAM [9,74,75. Moreover, hemocysteine, the intermediate product of the methionine cycle, can produce glutathione (GSH) through cystathionine and cysteine in the transsulfuration pathway [73]. Collectively, SGOCP, the main method of serine utilization, provides an integration point for cellular metabolism, contributing to diverse biological functions by converting serine and glycine into several metabolites.

Although serine catabolism enzymes exist in both the cytoplasm and mitochondria, most serine is transported to mitochondria through sideroflexin 1 to generate formate for use in cytosolic nucleotide synthesis [76]. However, a recent study has shown that folate availability determines whether serine is decomposed by the cytoplasmic or mitochondrial pathway. In cells with low expression of solute carrier family 19 member 1, encoding the reduced folate carrier, serine catabolism occurs mainly in the cytosol catalyzed by SHMT1. In the presence of high intracellular folate concentrations, serine is used in mitochondria by SHMT2, MTHFD2, or methylenetetrahydrofolate dehydrogenase 1 like [77]. However, under the condition of physiologic folic acid, the cytosol and mitochondrial 1C metabolic flux in a series of cancers were analyzed. This study challenges the previous view that mitochondrial folate metabolism is the only contributor to 1C units in tumors.

#### The TCA pathway

Inhibition of the SSP can reroute glucose-derived carbon into the TCA cycle and increase the flux of the TCA cycle [21]. Until now, the conversion of glucose-derived carbons into TCA cycle intermediates under PHGDH inhibitor treatment has not been addressed in detail. However, the SSP contributes ∼50% of the total anaplerotic flux of glutamine into the TCA cycle [21]. Reid et al. [78] proposed that elevated PHGDH concentration contributes to nucleotide metabolism mainly through the TCA cycle and pentose phosphate pathway. Correspondingly, in triple-negative breast cancer cells with high isocitrate dehydrogenase 2 expression, PHGDH and PSAT1 knockout can lead to impaired TCA cycle entry [79].

Therefore, cancer cells shunt carbon from the glycolytic pathway to the serine pathway to reduce carbon flux in the TCA cycle. The increased serine pathway can in turn promote the TCA cycle by supplementing the intermediates in the TCA cycle. The formation of such a complex pathway suggests that the serine pathway is necessary for tumor cell growth. Hence, regulating the TCA cycle from the SSP pathway is a promising approach for the treatment of tumors.

#### Lactic acid synthetic pathway

Lactic acid in the tumor microenvironment (TME) is associated with diverse cellular processes, such as tumor angiogenesis, invasion, metastasis, macrophage polarization, and T-cell activation [[80], [81], [82]]. Low expression of PHGDH in Sertoli cells of varicocele patients can lead to reduced lactate production by affecting glycolysis [22]. Meanwhile, lactic acid can reduce the production of glucose-derived serine in proliferating T cells by consuming NAD^+^ [83]. Although there is no direct evidence of a link between serine biosynthesis and lactic acid formation in tumors, the relationship deserves to be further investigated.

#### Lipid synthetic pathway

**FIGURE 2:**
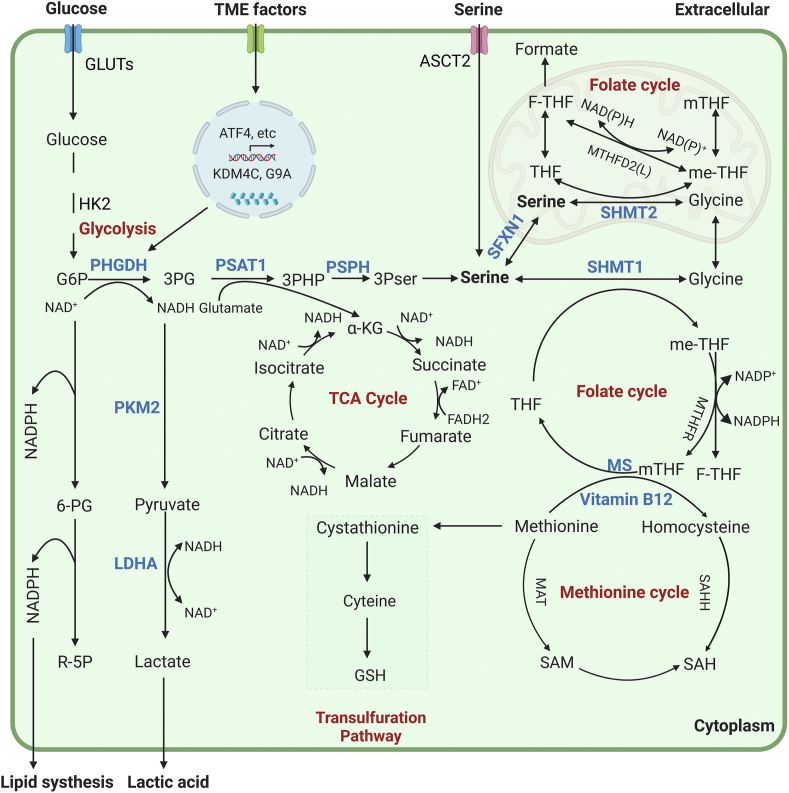
The serine anabolic and catabolic pathway. Serine can be synthesized de novo by cells (glycolysis and catalytic enzyme pathway) or absorbed through the transporter alanine–serine–cysteine transporter (ASCT)2. De novo synthesis of serine is based on glycolysis or the gluconeogenic intermediate 3-phosphoglyceric acid (3PG), which is divided into 3 steps: *1*) phosphoglycerate dehydrogenase (PHGDH) catalyzes the first step of NAD^+^-dependent oxidation of 3PG to 3-phosphohydroxypyruvate (3PHP); *2*) phosphoserine aminotransferase 1 (PSAT1) then converts 3PHP into 3-phosphoserine (3PS) in a glutamate (Glu)-dependent transamination reaction; and *3*) phosphoserine phosphatase (PSPH) catalyzes the last step of serine synthesis through hydrolysis of 3PS. The resulting serine can be decomposed in mitochondria or cytoplasm. In mitochondria, serine in the cytoplasm is transported into mitochondria by SFXN1 and catalyzed by SHMT2 to form glycine and 1-carbon units. The latter combine with tetrahydrofolate (THF) to form methylene tetrahydrofolate (me-THF) and enter the folic acid cycle. In the mitochondrial cycle, 1-carbon units are converted into formate that enters the cytoplasm. This cycle is accompanied by the production of NAD(P)H. In the cytoplasm, serine in the cytoplasm is catalyzed by SHMT1 to produce one-carbon units that enter the folate cycle and produce NADPH for lipid synthesis. The cytoplasmic folate cycle is coupled to the methionine cycle, which generates methyl groups for cellular biosynthesis and posttranslational modifications. The sulfur from homocysteine, which is generated from the methionine cycle, can be transferred to serine to form cystathionine by the action of cystathionine β-synthase (CBS) and is further converted to cysteine.

Serine has been shown to promote the growth of a variety of tumors through the lipid metabolism pathway (sphingolipids, phospholipids, and ceramide). Under the condition of serine deprivation, serine palmitoyltransferase can catalyze alanine to form deoxysphingolipids and thus slowdown colorectal tumor growth [12]. Similarly, serine restriction slows ceramide synthesis, leading to mitochondrial fragmentation and thereby inhibiting breast tumor cell proliferation [84]. Recent studies proposed that NADPH from folate-mediated serine catabolism is involved in fat synthesis in the liver and adipose tissue by the SHMT1–MTHFD1–aldehyde dehydrogenase 1 family member L1 axis [85]. Serine supplementation supports liver ceramide synthesis and is important to for maintaining lipid homeostasis in liver tissue [86]. Conversely, serine inhibition contributes to the overaccumulation of hepatic lipids and dysregulation of liver lipid metabolism in the mouse liver, which is a hallmark of nonalcoholic fatty liver disease [87,88]. Generally, serine is associated with several metabolic pathways and the synthesis of various substances in cancer cells and cancer-associated cells. Therefore, it is of great significance to study its regulatory factors, metabolites or effectors, signaling pathways, and function in cancer (Figure 2).

### Serine Metabolism and Tumorigenesis

It has long been known that both endogenously synthesized and exogenously ingested serine are associated with cancers and functionally support cancer development [9,13,25,36,89]. Disrupting serine synthesis can be detrimental for some tumors because decreasing PHGDH expression impairs the growth of subcutaneous lung cancer [90] and breast cancer xenografts [91,92]. However, PHGDH knockdown does not affect tumor growth in a different breast cancer model [93], arguing that SSP activity is only required in some contexts. Beyond the SSP, serine dietary provides a proliferative advantage to melanoma and breast cancer, but it is argued that serine availability is low in tumors arising in these tissues. Of note, serine availability does not seems to be limiting in all tumors; some breast cancers express SSP enzymes at low levels [92] and are not sensitive to loss of pathway activity [91,94]. In addition, a recent study reported that brain metastases rely on PHGDH to fulfill their biosynthetic needs for nucleotides because of the limited serine availability (28 μM) in this particular environment [31]. Rinaldi et al. [95] discovered that lung metastases, but not the corresponding primary breast tumors, rely on PHGDH to elevate the pyruvate-driven activity of serine biosynthesis, resulting in increased sensitivity to the mTORC1 inhibitor rapamycin. These findings indicated that the metabolic and nutrient requirements and availability to activate growth signaling differ between the primary cancer site and the metastatic niche.

**FIGURE 3:**
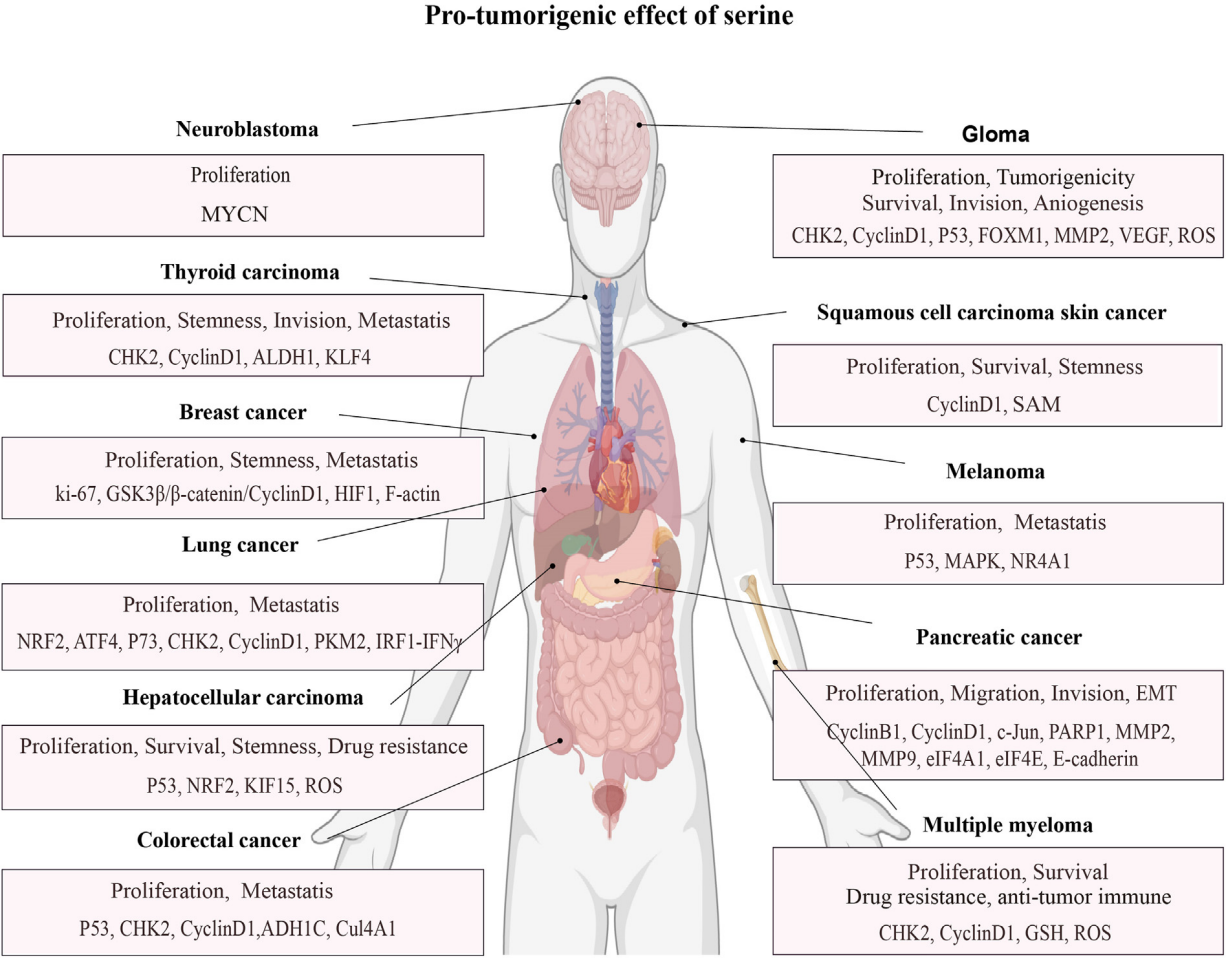
Protumorigenesis effect of the serine metabolism pathway. Serine functions as a powerful tumor promoter in most cancers. Enhanced serine signaling subsequently drives several oncogenic processes—cell proliferation and survival: checkpoint kinase 2 (CHK2), cyclinD1, tumor protein p53 (p53), nuclear factor erythroid 2-related factor 2 (NRF2), activating transcription factor 4 (ATF4), tumor-related protein p73, MYCN, FOXM1, growth factor [insulin-like growth factor (IGF)-1, epidermal growth factor (EGF), and EGF receptor]; stemness: hypoxia-inducible factor (HIF)-1, actin family member 15 (KIF15), S-adenosylmethionine (SAM); EMT progression: E-cadherin; angiogenesis, vascular endothelial growth factor (VEGF), EGF, fibroblast growth factor 2 (FGF2), CXCL1, interleukin (IL)-8, and IL-1; invasion and metastasis: matrix metallopeptidase (MMP; MMP2 and MMP9), VEGF, EGF, fibroblast growth factor 2 (FGF2), nuclear receptor subfamily 4, group A, member 1 (NR4A1), CXCL1, IL-8, Cul4A1, ADH1C, PKM2, and IRF1-IFNγ; and drug resistance: glutathione (GSH) and reactive oxygen species (ROS).

Generally, serine metabolism has been implicated in all stages of tumorigenesis, including tumor initiation, promotion, and progression, where it modulates cell proliferation, stemness, epithelial-to-mesenchymal transition (EMT), angiogenesis, and metastasis (Figure 3). Consequently, understanding the role of serine metabolic reprogramming in tumors is helpful for cancer treatment.

### Tumor initiation

Tumor initiation is mainly induced by the occurrence of multiple oncogenic alterations, which provides tumor-initiating cells with advantageous proliferation and survival properties by activating the serine catabolism pathway [8,96]. Tissue stem cells are one of the cells of origin for many malignancies. Baksh et al. [25] proposed that extracellular serine can control the balance between epidermal stem cell self-renewal and differentiation in squamous cell carcinoma (SCC). When extracellular serine is limited, epidermal stem cells activate de novo serine synthesis, which in turn setimulates α-ketoglutarate (KG)-dependent dioxygenases and activates differentiation programs partly through H3k27me3. Accordingly, serine starvation or enforced α-KG production antagonizes SCC growth [25]. This indicates that DNA methylation induced by serine contributes to tumor initiation.

DNA methylation provided by serine-elicited SAM through the SGOC pathway, particularly the methylation silencing of tumor suppressor genes or genes related to differentiation, is often related to the occurrence of tumors [74]. The increase in mutagenic targets and the accumulation of oncogenic mutations drive tumor initiation through the acquisition and proliferation of stem cells [97]. Moreover, accumulation of intracellular serine contributes to aberrant control of the cell cycle and promotes cancer stemness acquisition by increasing the number of DNA-damaged cells, which drives carcinogenic mutations and tumorigenesis [30,98]. These results increase the possibility that targeting serine metabolism is a promising therapeutic avenue to eliminate oncogenic stem cells.

### Tumor promotion

Tumor promotion is characterized by enhanced cell proliferation and survival of tumor cells or progenitors that contribute to the development of primary tumors [99,100]. High proliferation efficiency means that a large number of macromolecules are needed. Increased serine concentrations can provide raw materials for the synthesis of nucleotides, proteins, lipids, and other macromolecules required and maintain redox state homeostasis for the rapid proliferation of tumor cells [10]. One mechanism by which malignant cells ensure their proliferation and survival is by inactivating tumor suppressor function. Compelling evidence proves that cell cycle regulators and tumor suppressors play a critical role in cellular homeostasis [101,102]. Accumulation of intracellular serine contributes to aberrant control of the cell cycle and promotes cell proliferation and growth by inducing the expression of cell cycle checkpoint kinase 2 and cyclin D1, thereby driving tumor promotion [98,103]. Moreover, serine and 1-carbon metabolism link mTOR signals to DNA methylation, resulting in oncogene mutations that provide continuous activation of growth factor [insulin-like growth factor (IGF)-1, epidermal growth factor (EGF), and EGF receptor] signals for cancer cells. Moreover, mTORC1 and mTORC2 regulate the expression of a series of glucose uptake–related and glycolysis-related genes (GLUT1 and phosphofructokinase, platelet), thus promoting de novo serine synthesis and cell proliferation and survival [74,104]. Recent evidence has shown that the overexpression of PHGDH prevents ubiquitination of the oncogenic transcription factor forkhead box M1 and upregulates the expression of vascular endothelial growth factor (VEGF), matrix metallopeptidase 2, cell cycle checkpoint kinase 2, and cyclin D1, which promotes the proliferation, migration and invasion of glioma cells [98,105]. Glucose restriction induces the phosphorylation of PHGDH by p38 at Ser371 and promotes the translocation of PHGDH from the cytosol into the nucleus. The altered PHGDH activity restricts NAD^+^ concentrations and compartmentally facilitates pancreatic cancer cell proliferation and growth by inhibiting NAD^+^-dependent poly(ADP-ribose) polymerase 1 activity for the poly(ADP-ribosyl) action of c-Jun [106]. In addition, PSAT1 activates the glycogen synthase kinase 3β/β-catenin pathway in estrogen receptor (ER)-negative breast cancer to increase the expression of cyclin D1 and promote cell proliferation [107]. Furthermore, activation of the SSP pathway reduces ROS production and promotes the survival of tumor cells under hypoxic conditions or glucose deficiency [28,108].

However, because serine acts as a necessity for multiple metabolic pathways, it can also become rate limiting for growth and survival for several types of cancers. Banh et al. [109] found a subset of human pancreatic ductal adenocarcinoma cell lines lacking expression of serine biosynthesis pathway (SBP) enzymes (e.g., PHGDH) that were dependent on exogenous serine for growth and demonstrated that targeting the recruitment of neuronal axons releases amino acids, such as serine, which was able to rescue the growth of exogenous serine–dependent pancreatic ductal adenocarcinoma cells in Ser/Gly-deprived conditions [109].

### Tumor progression

Malignant progression is fueled by enhanced cancer cell proliferation, oncogenic mutation accumulation, and suppressed cell apoptosis. Serine metabolism not only contributes to the malignant phenotype but also triggers cancer stemness, angiogenesis, EMT, and metastasis [110,111].

#### Tumor stemness

Cancer stem cells have been implicated in metabolism, progression, and recurrence [[112], [113], [114]]. A growing amount of evidence links SSP with cancer stem cells, as demonstrated by Samanta et al. [115,116], who showed that PHGDH is required for maintaining breast cancer stem cells induced by hypoxia. Sharif et al. [117] demonstrated a novel link between PHGDH and stemness-maintaining transcription factors POU class 5 homeobox 1, nanog homeobox, SRY-box transcription factor 2, KLF transcription factor 4, and lin-28 homolog B in cancer stem-like cells, such as glioblastoma, lung carcinoma, breast carcinoma, embryonal carcinoma, breast cancer, and brain cancer. Moreover, PHGDH activity is directly related to enhanced tumor cell stemness by controlling the SRY-box transcription factor 2–OCT4 master complex of stemness in thyroid cancer [118]. PHGDH inhibition increases the ubiquitination and degradation of OCT4 to reduce stemness through posttranslational modifications [117]. In addition, by interacting with actin family member 15, PHGDH prevents its own degradation and promotes the phenotype and malignant transformation of cancer stem cells (CSCs) through ROS imbalance in HCC [119].

In addition, HIF-1 has been reported to mediate serine synthesis and mitochondrial 1-carbon (folate cycle) metabolism to increase mitochondrial antioxidant production (NADPH and GSH) [60]. Dynamic maintenance of ROS homeostasis is required for induction of the breast cancer stem cell phenotype [116]. Therefore, serine catabolism plays an important role not only in “biomass accumulation” in most cancers but also in malignant transformation of CSCs and tumor progression [60].

#### Tumor angiogenesis

For tumors, particularly solid tumors, the formation of new blood vessels is an important link in tumor progression, metastasis, and drug resistance. As tumors mature, tumor cells secrete various angiogenic factors, such as VEGF, EGF, fibroblast growth factor 2, C-X-C motif chemokine ligand 1, and IL-8 [120,121]. These factors regulate endothelial cell proliferation and tube formation, consequently forming blood vessels that supply nutrients and oxygen to the tumor mass [122].

Serine plays a crucial role in tumor angiogenesis induced by growth factors and chemokines. Studies have found that PHGDH increases the expression level of VEGF to enhance the progression of glioma brain tumors [98]. However, the specific mechanism by which PHGDH regulates glioma angiogenesis has not yet been reported. Recent studies have proposed that human umbilical vein endothelial cells rely on the SSP for hemin synthesis to maintain mitochondrial respiration and homeostasis. Supplementation of hemin in PHGDH^KD^ ECs restored electron transport chain function and rescued apoptosis and angiogenesis defects [110]. Unfortunately, there is little research on how serine promotes angiogenesis in tumors, and this field needs further exploration.

#### Tumor invasion and metastasis

Tumor progression is driven by local invasion and distant metastasis of transformed cells through blood and lymph vessels. Tumor cells acquire their invasive properties through a process known as EMT, which is characterized by reduced expression of epithelial markers such as E-cadherin [123,124].

Numerous studies have shown that the SSP pathway is closely related to the metastasis of breast cancer, lung cancer, colon cancer, pancreatic cancer, and thyroid cancer [31,95,116]. Soflaee et al. [125] revealed that a decrease in purine nucleotides inhibits PKM2 activity, causing glucose-derived carbon to be channeled from glycolysis into serine biosynthesis. Augmented serine/1-carbon metabolism is accompanied by stimulation of an EMT program, which promotes the invasive ability and dissemination of cancer cells (e.g., A375, SK-MEL-28, LNCaP, CAL-51, A549, B16, and HeLa) [125]. PHGDH promotes EMT, invasion, and distribution to distant organs by inhibiting the expression of E-cadherin in cancer cells and increasing chemokines in cancer-associated fibroblasts [126,127]. Moreover, PHGDH monoubiquitinated by CUL4A, resulting in an increase in SAM expression to upregulate the expression of cell adhesion genes (laminin subunit γ2 and cysteine-rich angiogenic inducer-61), further promoting the metastasis of colon cancer [64]. In addition, PSAT1 and PSPH, which catalyze the final and irreversible step of serine synthesis, promote tumor metastasis independent of their serine synthase activity. For example, increased PSAT1 promotes the metastasis of lung adenocarcinoma by inhibiting the interferon regulatory factor 1–interferon γ pathway [128]. In EGF receptor–activated lung cancer, overexpression of PSAT1 contributes to tumor cell metastasis by promoting nuclear PKM2 translocation [19]. PSAT1 also promotes breast cancer cell metastasis through F-actin cytoskeleton rearrangement and cell morphology [129]. Correspondingly, PSPH not only promotes tumorigenesis and metastasis through the SSP in breast cancer and colon cancer [130] but also facilitates melanoma growth and metastasis by increasing nuclear receptor subfamily 4, group A, member 1 expression [26]. Generally, these studies show that serine metabolism plays an essential role in cancer stemness, EMT, invasion, and metastasis. The combination of purine and serine synthesis inhibitors could have potential as a therapeutic strategy to hamper both cancer cells growth and invasive migration. More studies dissecting the mechanisms by which serine metabolism regulates these processes are necessary.

### Serine Metabolism and Tumor Immunity

**FIGURE 4:**
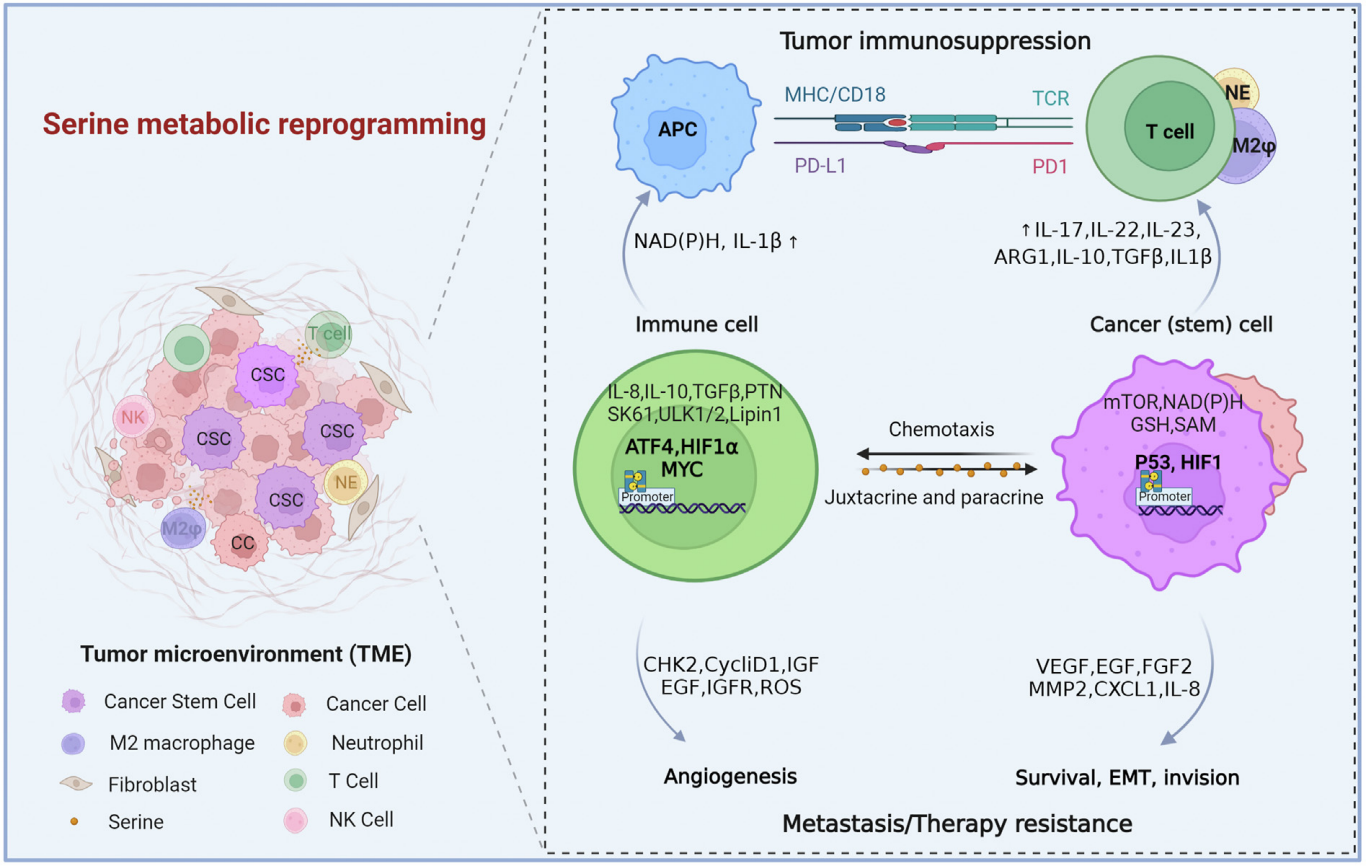
Serine signaling allows crosstalk between cancer cells and immune cells. Serine activation in cancer cells then activates signaling pathways that promote cancer cell proliferation and survival, epithelial-to-mesenchymal transition (EMT), invasion, angiogenesis, metastasis, and drug resistance. Cancer cells can recruit more immune cells to the tumor microenvironment by producing cytokines and chemokines that promote tumorigenesis and metastasis. Serine activation in immune cells induces the production of cytokines, chemokines, growth factors, and proteinase such as vascular endothelial growth factor (VEGF), endothelial growth factor (EGF), fibroblast growth factor (FGF) 2, matrix metalloproteinase (MMP)-2, interleukin (IL)-8, and CXCR1. Serine activity promotes the expression of IL-1β and NAD(P)H by antigen-presenting cells (APCs), thereby preventing their maturation and compromising T cell tumor immunosuppression. The production of ARG1, IL-10, TGF-β, IL-1β, IL-17, IL-22, and IL-23 in cancer (stem) cells strengthens the immunosuppressive network, contributing to tumor growth.

Since its discovery, serine has emerged as an essential modulator of immune component generation and function and immune homeostasis [36,131]. The balance between the tumorigenic and antitumor immunity function of serine metabolism is dictated by the abundance and activation state of distinct cell types and by the expression profile of various immune mediators and modulators in the TME [122]. TME consists of diverse cellular constituents, such as innate immune cells (macrophages, dendritic cells, neutrophils, and natural killer T cells), adaptive immune cells (T cells and B cells), mast cells, myeloid-derived suppressor cells, cancer cells, and surrounding stromal cells (fibroblasts, endothelial cells, pericytes, and mesenchymal cells) [99,122]. These heterogeneous cells act in autocrine and paracrine mechanisms to communicate with each other by directing contact or cytokine and chemokine signaling and modulating tumor progression. Generally, serine serves as a crucial oncogenic metabolite that contributes to immune cell generation, recruitment, and function (Figure 4) [37].

### Myeloid cells

Tumor-associated macrophages (TAMs) are classified into 2 reversible phenotypes—proinflammatory M1-type macrophages and protumoral M2-type macrophages. Functionally, M2-type macrophages release immunosuppression factors such as IL-10, transforming growth factor (TGF)-β, and arginase 1, to exert immune effects and release stem cell inducers, such as IL-8, IL-10, IGF, TGF-β1, and pleiotrophin, to maintain cancer stemness [[132], [133], [134], [135], [136], [137], [138], [139]]. Serine serves as an essential regulatory factor of macrophage polarization, and its gene deletion or pharmacologic inhibition induces the repolarization of TAMs from the protumorigenic M2 phenotype to the tumoricidal M1 phenotype [140,141]. Moreover, research has found that serine is a major contributor to α-KG production and M2 polarization in macrophages through the tuberous sclerosis complex-mTORC1 pathway to affect the progression of malignancies [141]. Furthermore, the activation of PSAT1 induced by a protein kinase RNA-like estrogen receptor kinase-signaling cascade in macrophages promotes serine biosynthesis, induces M2-type macrophage polarization by producing α-KG, and enhances the efficacy of immune checkpoint programmed cell death protein 1 in melanoma [140]. Moreover, inhibiting both endogenous and exogenous serine metabolism promoted the expression of IGF-1 thorugh a reduction in SAM-mediated histone H3K27me3. Elevated IGF-1 activates the p38-dependent Janus kinase–signal transducer and activator of transcription (STAT)1 axis, thus promoting M(interferon-γ) polarization and inhibiting polarization toward the STAT6-mediated M(IL-4) phenotype [142]. These results revealed a new mechanism by which serine metabolism orchestrates macrophage polarization.

The activation of SSP contributes to the acquisition of malignant characteristics in mammary epithelial cells induced by macrophage immune infiltration [143]. Recently, Shen et al. [144] demonstrated that virus-infected macrophages display decreased expression of SSP enzymes, which induces the expression of the V-ATPase subunit ATP6V0d2 by inhibiting SAM-dependent H3K27me3 occupancy. This finding suggests that targeting serine metabolism may be a therapeutic strategy against virus-infected macrophages. However, growing evidence has shown that TAMs have a crucial role in tumor development, metastasis, angiogenesis, TME remodeling, and treatment response [[145], [146], [147]]. Thus, the regulation of macrophage-dependent serine metabolism can be used as an auxiliary strategy for tumor therapy.

### Lymphocytes

T and B cells are the most prominent and potent antitumor immune regulators [[148], [149], [150]]. Serine can produce a variety of metabolites through SGOCP, which affects the development, proliferation, and differentiation of T lymphocytes [CD8^+^ T cells, effector T cell (T_EFF_), and regulatory T cells] and B lymphocytes, indicating that altered serine pathways might compromise T cell–mediated antitumor immunity [37]. Ma et al. [32] proposed that serine regulates the expansion of cloned T cells by supporting 1-carbon metabolism for the de novo production of purine nucleotides. Similarly, GSH-deficient regulatory T cells display increased serine synthesis, mTOR activation, and proliferation, which is linked to both autoimmunity and increased tumor rejection in vivo [151]. In addition, Epstein-Barr virus upregulates the import and synthesis of serine to augment 1C flux, which drives B cell proliferation [152]. These results indicated that serine, as an immunomodulator, can shape adaptive immunity by influencing the proliferative capacity of T and B cells. However, the relationship between serine activity in T cells and B cells and tumor immunity is still uncertain. We can only speculate that serine activity blocks the infiltration and elicits the exhaustion of T or B cells. Future studies should focus on exploring the role of serine activity in T cell and B cell tolerance, immune editing, and antitumor immunity in the TME.

In addition, serine biosynthesis uses NAD^+^, an important cofactor of the redox reaction, to maintain cell homeostasis. NAD metabolism is based on crosstalk between cancer cells and immune cells, affecting the TME. The regulation of immune responses in the TME depends on different types of host cells, such as endothelial cells, mesenchymal stem/stromal cells, cancer-associated fibroblasts, and immune cells (e.g., lymphocytes, macrophages, natural killer cells, and neutrophils) [153,154]. However, growing evidence have shown that elevated serine has a crucial role in constructing tumor immune microenvironment. Although the underlying mechanism how serine orchestrates tumor-related immune cells remains nascent, targeting serine metabolism can be used as an auxiliary strategy for tumor immunotherapy.

### Serine-induced tumor immunosuppression

Cancer cells and immune cells rely on nucleic acid and protein synthesis, which are influenced by the cell nutritional status, such as the availability of amino acids. Serine can be converted into other kinds of amino acids and synthesize important macromolecular substances. It can also provide 1-carbon units for 1-carbon metabolism, which produces cofactors such as NADH, NADPH, and ATP to participate in multiple signaling pathways (e.g., Arg1, IL-10, and TGF-β) of tumor immunity [9,10,37,38,155].

Primordial T cells stimulated by tumor-specific antigens transform into an active state characterized by rapid proliferation to generate a T_EFF_ cell pool and mediate antitumor immunity [156]. The activation of T_EFF_ cells requires the heavy consumption of various nutrient factors, including serine, to support their proliferative demands and exert effector functions [156]. A recent study indicated that Epstein-Barr virus can augment 1-carbon flux by upregulating the import and synthesis of serine to maintain NADPH levels in infected cells, thereby increasing B cell proliferation and mediating antitumor immunity [152]. Furthermore, serine was reported to initiate 1-carbon metabolism to facilitate the generation of SAM, which can provide a methyl donor involved in the methylation of cellular DNA, RNA, and proteins to promote IL-1β production for tumor immunosuppression [157]. These results indicate serine-dependent metabolites, such as SAM, NADH, and NADPH, play a requisite role in regulation of tumor immunosuppression. Therefore, the clearance of these excessed product is extremely important to maintain the antitumor environments and enhance the efficacy of tumor immunotherapy.

In innate immunity, multiple innate immune cells, such as natural killer cells, T_EFF_ cells, and B cells, require mTOR (serine-threonine kinase)-promoting serine metabolism to maintain differentiation, growth and function [158]. Recent studies have shown that IL-23 induces the activation of mTOR in neutrophils, whereas blockade of the mTOR pathway inhibits IL-23–induced IL-17 and IL-22 production [159,160]. As a downstream element of mTOR, the upregulation of the transcription factor HIF-1α also augments the expression of *IL-17* and *IL-22* genes and contributes to the rapid proliferation of T_EFF_ cells [37,161]. The tumor suppressor menin prevents effector CD8^+^ T cell dysfunction by targeting mTORC1-dependent metabolic activation [162]. Conditional disruption of the mTORC1 coactivating protein in developing mouse B cells leads to a developmental block at the pre-B cell stage. This evidence suggests that accelerated serine catabolism is beneficial for the cloning and amplification of T cells and B cells, and altering mTOR is regarded as a coupler linking serine metabolism and antitumor immunity.

### Serine Metabolism and Its Potential Targets

Aberrant metabolic changes have always been the focus of tumor research and clinical therapy. Compelling evidence has shown that serine metabolic reprogramming in cancers is associated with treatment resistance, such as radiotherapy, chemotherapy, and immunotherapy resistance [30,163,164]. Understanding the importance of serine synthesis and catabolism in tumor cell growth and tumor immunity is beginning to provide new opportunities for tumor therapeutic intervention.

### Chemotherapy and radiotherapy resistance

Metabolic rewiring plays an essential role in drug resistance development. Emerging evidence has shown that serine metabolic reprogramming is considered a common metabolic feature of tumor cell resistance to doxorubicin, sorafenib, erlotinib, and 5-fluorouracil (5-FU), in addition to other chemotherapeutic agents [[18], [163]]. Triple-negative breast cancer cells exposed to doxorubicin undergo metabolic remodeling, resulting in increased serine synthesis regulated by PHGDH. Then, serine is converted into GSH, which counters doxorubicin-induced formation of ROS. Consequently, inhibition of PHGDH can increase the sensitivity of cells to doxorubicin [165]. Exposure of PHGDH-kd ER+ breast cancer cells to cytotoxic chemotherapy (carboplatin or doxorubicin) leads to increased mitochondrial ROS and blocks the enrichment of CSCs induced by chemotherapy [166,167]. Therefore, PHGDH may be a novel therapeutic target to reverse recurrence/resistance to tamoxifen therapy in ER^+^ breast cancer. In addition, PHGDH is a key factor in the resistance of HCC to sorafenib. Inactivation of PHGDH elevates ROS levels and induces HCC apoptosis on sorafenib treatment. Other Food and Drug Administration (FDA)-approved tyrosine kinase inhibitors have also been found, such as regorafenib or lenvatinib [29]. In myeloma and renal cancer, PHGDH is associated with bortezomib and sunitinib resistance [24,168]. Moreover, serine biosynthesis is also described as the mechanism of intrinsic and acquired drug resistance to vemurafenib in non–small-cell lung cancer, pancreatic cancer, and melanoma [169]. Obviously, increased serine biosynthesis is positively associated with tumor drug resistance and predicts a poor survival prognosis, and further studies will be needed to elucidate the exact mechanism.

Recent studies have shown that PHGDH is upregulated in Ras mutant tumor patient-derived xenografts of the acquired MAP/ERK kinase–resistant inhibitor PD901. Inhibition of PHGDH can resensitize drug-resistant cells to MAP/ERK kinase inhibitors or vemurafenib [170]. Similarly, PHGDH is also a key driver of acquired resistance to erlotinib in lung adenocarcinoma (LUAD) [171,172]. In addition, inhibition of serine biosynthesis and dietary restriction have been proven to enhance the antitumor activity of 5-FU by inhibiting PAST1 expression [30]. Therefore, serine disruption is a promising strategy to overcome tumor drug resistance [172].

Previous studies have demonstrated that serine biosynthesis can enhance redox balance and nucleotide synthesis, thereby reducing radiosensitivity. The activation of serine biosynthesis is related to radiotherapy resistance in head and neck SCCs, and more radiation-resistant cells have higher levels of serine metabolism [173]. Limiting serine in the diet can make cancer cells (e.g., colorectal, breast, and pancreatic cancer cells) sensitive to radiotherapy by inhibiting antioxidant reactions, nucleotide synthesis, and the TCA cycle [174]. Correspondingly, the inhibition of PHGDH can lead to increased radiosensitization of human colorectal cancer cells under hypoxia [164]. Another important aspect of serine metabolism is the ability to control stemness and self-renewal of stem cells. CSCs are key contributors to radioresistance in many tumor types, such as glioblastoma, head and neck SCCs, breast cancer, and pancreatic cancer [163,175,176]. In summary, this evidence indicates that inhibition of the serine metabolic pathway (e.g., PHGDH) is a potential target to improve chemotherapy and radiotherapy effects.

### Dietary restriction and targeting inhibitors

The serine biosynthesis pathway may be a relevant target to improve the outcome of cancer patients who receive chemotherapy, including dietary restriction. Both endogenous and exogenous serine restriction contribute to impaired tumor resistance to 5-FU or other treatments. Moreover, the use of PHGDH inhibitors can increase the sensitivity of cancer cells to chemotherapy drugs. Treatment with the PHGDH inhibitor NCT-503 works synergistically with sorafenib to abolish HCC growth [29]. The use of second-generation PHGDH inhibitors (PH-719 and PH-755) inhibits the growth of breast cancer and colon cancer cells in serine-limited environments and attenuates breast cancer brain metastasis [11,31]. Therefore, it is critical to use inhibitors of PHGDH to treat patients with high PHGDH expression or *PHGDH* gene amplification.

To date, a number of PHGDH inhibitors have been reported and can be divided into 2 main types according to their binding site: allosteric and orthosteric inhibitors. The allosteric inhibitors include CBR-5884, disulfram derivatives, piperazine-1-carbothioamide scaffold, α-ketothioamide scaffold, pyrazole-5-carboxamide derivatives, PKUMDL-WQ, and natural products azacoccone E and ixocarpalactone A. The orthosteric inhibitors contain indole amide derivatives, phenylpyrazole-5-carboxamide derivatives, and some fragment hits [33,177].

Unfortunately, no PHGDH inhibitors have yet entered clinical studies. Although known allosteric inhibitors have good effects on cancer cell lines in vitro and xenotransplantation models in vivo, their effects are not as good as those of orthosteric inhibitors in enzymatic evaluation [34]. This disconnection is difficult to explain because the exact binding sites of many allosteric inhibitors are unclear or predicted only from docking studies. The reported orthostatic inhibitors showed a strong activity in the determination of the enzyme activity, but their cellular efficacy was poor. Notably, inhibition of PHGDH led to compensation of other metabolic pathways, resulting in drug resistance to PHGDH inhibitors. Consequently, it is necessary to further study the inhibition of the serine metabolic pathway to better understand its potential effect on other pathways, and it is of great significance to find new inhibitors with novel structures, strong activity, and good pharmacokinetics. Recently, proteolysis-targeting chimeras has been developed as a useful technology for targeted protein degradation [178]. This strategy might potentially be used to achieve efficient degradation of the PHGDH protein in a quick and direct manner.

### Immunotherapy potential

Immunotherapy that manipulates the TME to exert antitumor immunity has recently attracted much attention. Serine has been identified as a promoter of myeloma progression by controlling M2 macrophage polarization to exert antitumor immune activity. Inhibition of serine synthesis through gene deletion or pharmacologic inhibitors promotes transformation from the protumorigenic M2 phenotype to tumoricidal M1 macrophages in a model of drug-resistant relapsed/refractory myeloma [140]. Moreover, serine biosynthesis produces α-KG to polarize M2-type macrophages and enhances the efficacy of the immune checkpoint programmed cell death protein 1 in melanoma [141]. Thus, the inhibition of serine activity to suppress the tumor-promoting microenvironment can be coupled with chemotherapy and immunotherapy to enhance therapy effectiveness and control tumor relapse [87]. In addition, the limitation of serine also affects the fate of normal cells, which means that in healthy cells, the limitation of serine synthesis produces some predicted consequences [25]. Therefore, targeted serine metabolism from the laboratory to clinical treatment of tumors still has a long way to go, and there are still many difficulties to overcome.

### Perspective and Conclusion

**FIGURE 5:**
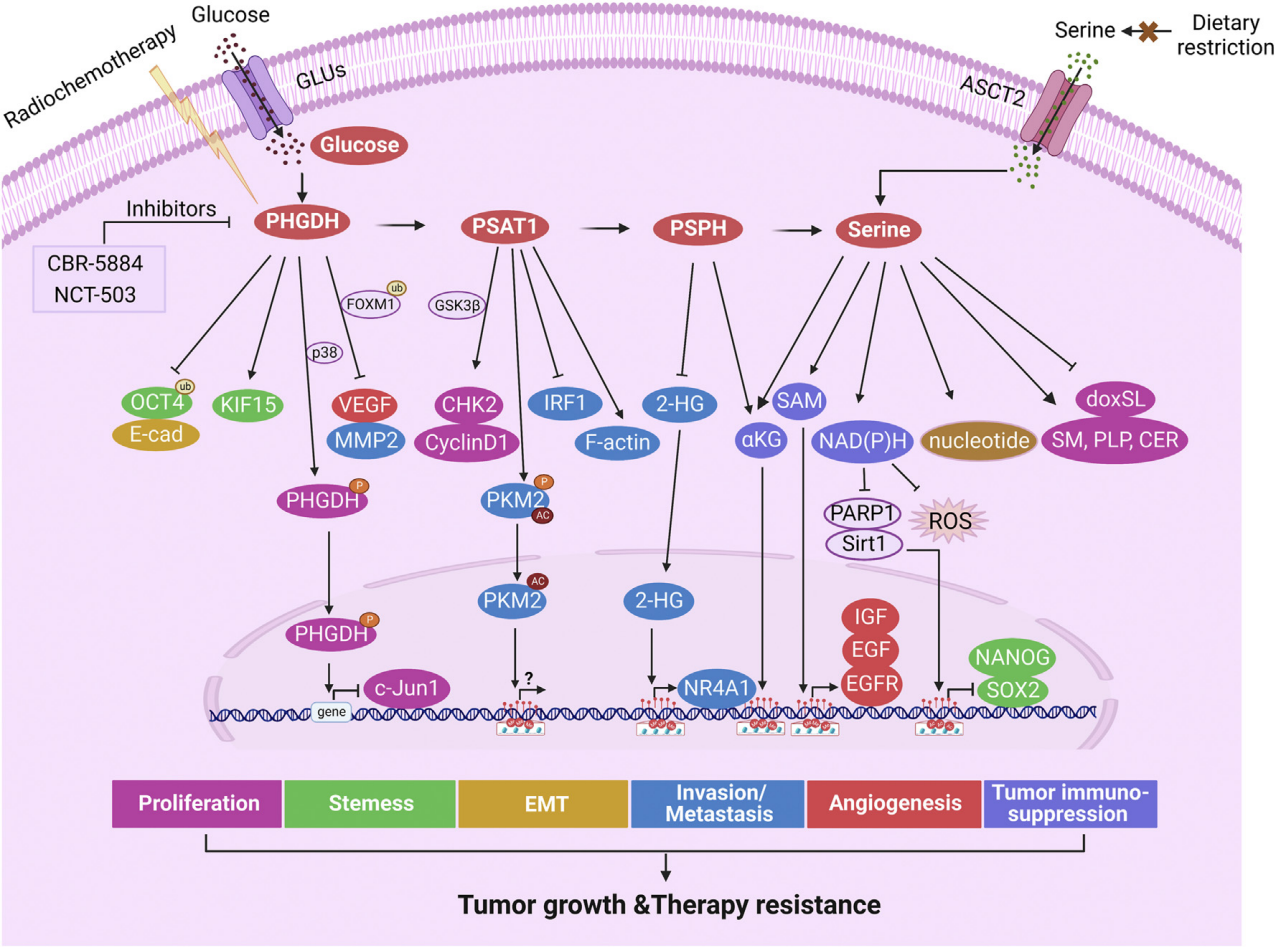
Serine metabolism drives tumorigenicity and drug resistance. Serine can be synthesized by PHGDH, PSAT1, and PSPH or absorbed by ASCT2. PHGDH inhibits FOXM1 ubiquitination and promotes the expression of CHK2 and cyclin D1 to accelerate cell proliferation. The phosphorylation of PHGDH by p38 at Ser371 leads to its entry into the nucleus, thus facilitating cell proliferation. PHGDH enhances tumor cell stemness by inhibiting OCT4 ubiquitination, binding to KIF15, or producing NADPH. PHGDH accelerates EMT by inhibiting E-cadherin. PHGDH promotes the expression of VEGF by inhibiting FOXM1 ubiquitination and the expression of IGF, EGF, and EGFR to facilitate tumor angiogenesis. PHGDH promotes MMP2 expression by inhibiting FOXM1 ubiquitination. PSAT1 blocks the IRF1-IFNγ pathway and promotes cytoplasmic PKM2 phosphorylation and acetylation, leading to PKM2’s translocation and interaction with PSAT1 and affecting rearrangement of the F-actin skeleton. PSPH inhibits 2-HG to promote the transcription of NR4A1. α-KG–catalyzed and PSPH-catalyzed reactions affect H3K27me3. The occurrence of the SSP pathway can produce cofactors such as SAM and NAD(P)H for ROS stabilization and promote the synthesis of intracellular nucleotides for DNA damage repair. The NAD^+^:NADH ratio affects PARP1 and sirt1, which are related to the biological behaviors of tumor cells, such as proliferation, stemness, EMT, and immunity. All these processes promote tumor growth and therapy resistance. Moreover, restriction of dietary serine and PHGDH-targeted inhibitors in combination with radiochemotherapy can achieve good therapeutic effects.

Serine has been identified as a metabolic linchpin that connects glycometabolism, tumorigenesis, tumor immunity, and clinical therapy. This review summarized the underlying mechanisms and contribution of serine metabolic reprogramming in tumorigenesis, progression, and tumor immunity (Figure 5). Moreover, serine is depicted as a multifaceted tool that can be applied as a research target for understanding disease progression, a prognostic and predictive biomarker, and a potential therapeutic target in the fight against cancer.

Serine metabolism controls hubs of tumorigenic signaling, thereby providing an attractive strategy for targeting tumorigenesis, progression, and tumor immunity. However, there are fundamental gaps in our understanding of serine regulation, metabolic production, or effectors, signaling, and function, which currently makes it difficult to unequivocally determine the overall effect of the serine pathway and its clinical application in cancer.

First, to understand the events of serine metabolism more precisely, the heterogeneity and relationship of serine absorption, synthesis, and usage between cancer cells and cancer-associated cells merit further exploration. Recently, activated TAMs have been shown to have an increased capacity to consume glucose and produce surplus serine by responding to a specific local tumor environment [46]. Tissue-specific symbiotic effects between cancer cells and cancer-associated cells may elevate the reasonable distribution and usage of resources (e.g., glucose and amino acids), thereby facilitating the progression of tumor deterioration. Second, the mechanisms underlying tumorigenesis, progression, and tumor immunity events by serine metabolism, especially tumor stemness, immune response, and drug resistance, are still poorly understood. Further deciphering the cytological and molecular mechanisms of serine metabolism will contribute to more targets and strategies for cancer intervention. Finally, the complexity and diversity of serine metabolism in solid tumors (e.g., cancer types, stages, gene mutation and matrix stiffness) should be considered when selecting a serine-blocking therapeutic regimen, and these regimens should be tailored to the patient, cancer phenotype, and influence of the TME. It is important to overcome the above-mentioned challenges for clinically recognized applications targeting serine metabolism in treating cancer.

While most evidence supports the oncogenic function of serine, few studies have suggested the effect of immune cell expansion and antitumor immunity on serine metabolism. A plausible explanation to this paradox is the bifurcation of immune cell (T_EFF_, CD8^+^ T cell) metabolism. Glucose promotes cell bioenergetics and effector function, and serine metabolism acts as a metabolic checkpoint for nucleotide biosynthesis to control T-cell expansion without impacting bioenergetics or effector function. Strikingly, genes involved in serine metabolism are upregulated concomitantly with glycolysis in activated T cells, and their expression is largely restricted to early T_EFF_ cells undergoing rapid proliferation following antigenic encounter. However, the exhaustion of low antigen-affinity CD8^+^ T cells promotes the malignant progression of cancer. Future work will need to focus on understanding the roles of serine metabolism in T-cell recruitment, subsets, and activation state (i.e., primary versus memory T-cell responses) in tumors.

Generally, metabolic reprogramming, including serine metabolic reprogramming, is an important hallmark of malignant cancer. Many factors, including changes in metabolites, genetic factors, and microenvironmental physical cues, play a requisite role in the occurrence, development and therapy resistance of cancers. Metabolic regulation of serine is a basic but relatively unexplored area for understanding cancers. Both serine and serine metabolites have been proven to have an important impact on the formation and progression of tumors and treatment research. Further understanding of the role of serine metabolism in tumorigenesis, tumor immunity, and therapeutic applications will provide a platform for the development of more integrative and specific antitumor therapeutics.

## Funding

This work was supported by grants from the National Natural Science Foundation of China (grant numbers 12172072 and 11832008).

## Author disclosures

The authors report no conflicts of interest.
